# Integration of non-communicable diseases in health care: tackling the double burden of disease in African settings

**DOI:** 10.11604/pamj.2014.18.202.4086

**Published:** 2014-07-05

**Authors:** Florence Temu, Marcus Leonhardt, Jane Carter, Sylla Thiam

**Affiliations:** 1 African Medical and Research Foundation, International Training Centre, Nairobi, Kenya

**Keywords:** Non-communicable diseases, health care, burden of disease, Africa

## Abstract

Sub-Saharan African countries now face the double burden of Non Communicable and Communicable Diseases. This situation represents a major threat to fragile health systems and emphasises the need for innovative integrative approaches to health care delivery. Health services need to be reorganised to address populations’ needs holistically and effectively leverage resources in already resource-limited settings. Access and delivery of quality health care should be reinforced and implemented at primary health care level within the framework of health system strengthening. Competencies need to be developed around services provided rather than specific diseases. New models of integration within the health sector and other sectors should be explored and further evidence generated to inform policy and practice to combat the double burden.

## Introduction

Sub-Saharan Africa is experiencing a rapid epidemiological transition with the growing burden of non-communicable diseases (NCDs), as a result of the rapid urbanisation and westernisation of lifestyles, decreasing physical activity, changing dietary habits and increasing longevity of the population. In developing countries NCDs have now overtaken communicable diseases (CDs) as the prime cause of mortality (54% compared to 36%) [1, 2]. According to the World Bank, mortality due to NCDs will have risen from 28% in 2008 to 46% by 2030, representing a 64% increase. This will amount to a 34% increase in healthy years lost, based on Disability Adjusted Life Years [3]. A recent study conducted jointly by the World Economic Forum and Harvard University showed that NCDs will cost the world economy $47 trillion over the next 20 years, representing 75% of global gross domestic product (GDP) and surpassing the cost of the global financial crisis [4]. This is compared to an estimated cost of $11.4 billion a year in low- and middle- income countries to provide basic packages of cost effective strategies to prevent and treat NCDs. NCDs and CDs are therefore placing a joint strain on the already fragile health systems of Sub-Saharan African countries [5] with an expected adverse impact on the health of the people.

The Millennium Development Goals (MDGs) have proved to be a lost opportunity for addressing NCDs. NCDs strongly contribute to all eight MDGs but omission of NCD indicators in the MDGs has resulted in failure to secure adequate attention and donor funding for NCDs. This is linked to the global focus on communicable diseases (CDs), mainly HIV/AIDS, malaria and TB, which have been prioritized for major funding support over the last decade. In addition, vertical programming as the main approach to disease control has promoted fragmentation of the health services and placed additional constraints on the already limited human resources for health.

Despite the global agenda, a number of African countries have already put NCD policy frameworks into place; however, major challenges remain in the implementation of effective strategies. Improper resource allocation, poor priority setting and lack of effective and feasible integrative models are major constraints to implementing NCD strategies in African countries. This scenario clearly calls for urgent public health interventions for NCDs especially targeting productive age groups in developing countries.

In 2010, NCDs were formally recognized at the UN General Assembly as an important missing element in the Millennium Development Goals (MDGs) [6]. A meeting of the United Nations General Assembly in September 2011 addressed the prevention and control of NCDs. A number of resolutions were drawn up emphasizing the linkages between non-communicable diseases and communicable diseases, and a call made for appropriate integration. This paper seeks to analyse the issue of integration of CDs and NCDs in health care in developing countries and outlines the challenges and the need for innovative approaches to address the double burden of disease in Africa.

## Integration of Non-Communicable Diseases in African health systems

### 1. Integrative approaches for better health care

In the light of the rapidly increasing double-disease burden of CDs and NCDs in African countries, it is becoming essential to use the limited resources available in the most effective way. Health planners and policy and decision makers need to agree that health systems must address populations holistically and not address diseases in a discrete manner. Vertical disease programmes, with their targeted funding and interventions, have aimed to achieve specific results within a given time-frame, but at the expense of a holistic approach to managing health. On the other hand, an integrated approach seeks to address health problems by providing services in a comprehensive manner. These two approaches may not be mutually exclusive and ways of combining their respective strengths need to be explored in the context of health system strengthening [7]. According to the country specific context, approaches of integration could be considered on a case by case basis [8].

Knowing that interventions targeted against one of the disease burdens will impact the other, it is paramount that interventions are conducted jointly instead of competing for limited resources. This is a critical policy consideration requiring new thinking and approaches [9]. An approach towards health-centred management rather than a disease-centred model is now needed to tackle the two disease burdens in Africa. Integrative approaches provide holistic options focused on the health needs of people and communities, and thereby enhance community self-reliance [8]. This will give communities the opportunity to voice their concerns and provide inputs into interventions taking place in their home areas.

Given the scarcity of resources and competition in priority setting within the health sector and among other government sectors, there is clear justification for integration. All resources, both human and financial, need to be integrated to address the major disease burdens affecting communities. Integrated interventions are possible at all levels, including health facility, community and household, as long as they are well designed and packaged.

### 2. Strengthening Primary Health Care as part of Health System Strengthening

Knowing the critical role of strong health systems to deliver quality and timely services to meet targets, the World Health Organization has championed the concept of Health System Strengthening (HSS). This in turn has contributed to creating opportunities for donors to support HSS. Priorities for both CDs and NCDs should be used to address required improvements in the health system, addressing generic issues such as human resources, financing, drug supply, quality assurance and information management [10].

Prevention and control of NCDs are best implemented at Primary Health Care (PHC) level. It is therefore essential to build and strengthen health systems to effectively respond to the challenge of NCDs at this level [7, 11]. With much of disease management at primary health care level currently based on syndromic approaches, many early signs and symptoms of NCDs go unnoticed or are neglected. It is necessary for clinicians to revert to basic clinical practices including targeted history taking and physical examination, use of appropriate laboratory investigations to guide diagnostic and management decisions, and planning for follow-up and re-assessment. In addition, health workers have insufficient time and often lack the up to date knowledge required to counsel patients and community members on the lifestyle changes necessary to prevent and control NCDs.

Basic clinical and diagnostic skills are taught at pre-service level, but are usually not addressed in in-service training programmes, which are generally funded and organised by vertical disease-control programmes. In-service training programmes therefore rarely address an integrated approach to health care delivery. This requires a completely new approach to in-service training programmes, which should address enhancing the competencies of health personnel to address holistic health care needs of people and communities [8], organized around the scope of work that each health cadre performs [12]. Task shifting to lay health workers and communities may also be considered. Use of new technologies such as e-learning and m-learning could help train or retrain an important number of health workers in a short period of time [13].

African countries must avoid adopting the costly health care systems adopted by developed countries. Health systems in Africa were mainly designed to respond to CDs which affected millions of people, but the structure of health systems now need to evolve to address the nearly one billion of inhabitants who will live longer and suffer from a broader range of ailments. Frameworks and structures set up for CDs management such as supply chain management, training, communication, surveillance, supervision, and monitoring and evaluation need to be restructured to be able to address health problems in an integrated manner.

### 3. Models of integration

Many existing health interventions are already structured for ready integration, for example, antenatal care and family planning with holistic cervical cancer programming. Programmes for communicable diseases that have associations with NCDs can also be readily integrated, for example, HIV/AIDS and Tuberculosis (TB) with diabetes, HIV/TB/ Malaria and Maternal Health with cervical cancer and Kaposi.s sarcoma; malaria with Burkitt.s lymphoma; schistosomiasis with cancer of the urinary bladder.

Prevention packages for NCDs also lend themselves to integration. Addressing lifestyles and behaviour will also impact on the prevention of many communicable diseases, for instance, avoiding tobacco use and harmful use of alcohol, and eating a healthy diet will also promote a stronger immune system less prone to infection. Other health problems common in the developing world, such as nutrition and mycotoxicosis, will finally be addressed by health interventions delivered in an integrated manner.

Health systems will be better able to response to community health needs and priorities when people.s needs are central and there is proper use of community knowledge and participation. Preventive health interventions need to engage grassroots community and support groups instead of addressing individual patients on a one-to-one basis. Integration of NCD components can easily be included in comprehensive health education, communication and promotion packages for communities and families at household level. These can form an integral component of the work of community volunteers. Community-based disease surveillance and screening programmes can be used effectively for case finding, linked with effective referral systems and accessible health care that provides quality services properly equipped to address these conditions.

Given the challenges and scarcity of effective treatment centres for some non-communicable diseases, such as cancers, effective health referral systems need to be established at country level. With a clear vision for health and development under the leadership of regional economic bodies and/or the African Union, a pan-country mechanism can be used to create specialized Centres of Excellence for the prevention, care and research into specific diseases. This mechanism will also promote retention of locally available resources and could stimulate the development of medical tourism within Africa.

### 4. Evidence

Since for many years public health policies have focused on the control of infectious diseases, country-specific data on NCDs in Africa are scarce and most of the available data are based on estimations. Even national surveys such as Demographic and Health Surveys (DHS) do not provide data on NCDs. It is widely acknowledged that accurate information is crucial for informed policy decisions. Therefore monitoring and evaluation systems should be reinforced and integrated to gather data on non-communicable diseases [12]. Routine data can be complemented by health surveys to provide key information for decision-makers, programme planners and policy makers to prioritize the development and implementation of policies and strategies targeting NCDs.

Furthermore there are clear gaps in the evidence of successful implementation of NCD interventions within the primary health care setting in African countries. Models and approaches to properly inform the design of interventions that address the needs of communities and individuals are lacking [14]. There is an urgent need to fill these knowledge gaps to support the prevention and care of NCDs.

### Integration with other sectors: Multi-sectoral

Both CDs and NCDs have a negative effect on national income, productivity and household expenditure and therefore have an impact on economic growth. There is strong evidence that health is an important factor in economic development which calls for a global approach to address health for development. Therefore multi-sectoral action beyond the health sector is required. People with lower education and economic status are more vulnerable and are more exposed to ill health and are increasingly affected by NCDs.

Food security has become another important area for resource allocation for developing countries. Ensuring food security is critical for preventing NCDs, a major example being the link between aflatoxin exposure and several health-related conditions including acute and chronic aflatoxicosis, aflatoxin-related immune suppression, liver cancer, liver cirrhosis and maternal and child health problems such as anaemia, malnutrition and stunting [15].

Climate change and environmental degradation is another global agenda targeting developing countries. With the pace of urbanization and predominance of urban slums, urban planning and sustainable environment also need to be considered as one of the preventive aspects of NCDs. It is therefore important to address the determinants of health more holistically as an integrated component of comprehensive development policies to improve the health of populations. This will reduce inequalities and ultimately break the cycle of poverty and disease [5, 15, 16].

## Conclusion

Health profiles in the African context show that health care systems struggle to establish and maintain integrated policies and deliver a comprehensive range of essential primary care services, including promotion and prevention, early detection and timely and quality management of NCDs. As a result, a large proportion of people with high NCD risk remain undiagnosed and untreated. It is time to consider integrated models for inclusion of NCDs into interventions to address the double burden of disease in Africa and other developing regions of the world. Some NCD interventions can be readily integrated with minimal effort into practices and procedures already in place for control and prevention of communicable diseases. There is need to strengthen research to generate credible evidence for influencing policy and practice as health systems struggle to respond to the two burdens. Developing and testing models that guide health systems to be more responsive to the health needs of communities is essential. Global discussions for the Post-MDG 2015 agenda are clear opportunities to ensure NCDs are integrated and well embedded into the new goals and given the attention they deserve. There are number of initiatives taking place in Africa and globally such as Partnership for Aflatoxin Control in Africa (PACA) which need to be well articulated in the NCD agenda.
